# Protein Arrays: Issues to Be Addressed

**DOI:** 10.1002/cfg.105

**Published:** 2001-10

**Authors:** Michael J. Taussig

**Affiliations:** Chairman ESF Programme in Integrated Approaches to Functional Genomics The Babraham Institute Cambridge CB2 4AT UK

## Abstract

Protein arrays are fast becoming established as a means to monitor protein expression levels and investigate protein interactions and function. They present particular technical demands that will need to be solved in order to achieve the maximum capability of efficient and sensitive protein analysis in the high throughput setting of functional genomics. The following resumé of some major issues around this new technology was made as the chairperson’s introduction to the workshop session on peptide and protein chips.


**Comparative and Functional Genomics**
*Comp Funct Genom* 2001; **2**: 298–300.
**DOI:** 10.1002/cfg.105

**Conference Discussion**

### Protein arrays: issues to be addressed

### The chairperson's introduction to the session 'peptide and protein chips' from the ESF workshop 'Proteomics: focus on protein interactions'

Michael J. Taussig*
*Chairman, ESF Programme in Integrated Approaches to Functional Genomics, The Babraham Institute, Cambridge, CB2 4AT, UK*

*Correspondence to: M. J. Taussig, Chairman, ESF Programme in Integrated Approaches to Functional Genomics, The Babraham Institute, Cambridge, CB2 4AT, UK. E-mail: mike.taussig@bbsrc.ac.uk*

Received: 29 May 2001
Accepted: 6 August 2001

### Abstract

**Protein arrays are fast becoming established as a means to monitor protein expression levels and investigate protein interactions and function. They present particular technical demands that will need to be solved in order to achieve the maximum capability of efficient and sensitive protein analysis in the high throughput setting of functional genomics. The following resumé of some major issues around this new technology was made as the chairperson's introduction to the workshop session on peptide and protein chips. Copyright © 2001 John Wiley & Sons, Ltd.**

**Keywords:** **protein arrays; protein chips; proteomics; protein interactions; gene expression profiling; antibody array; scaffold; protein expression**

Array formats are well established for global analysis of nucleic acids, as in the use of oligonucleotide and cDNA arrays for gene expression profiling. In relation to protein expression, however, information from DNA arrays leaves much to be desired. They provide a relatively inaccurate guide to the final concentrations of gene products within the cell, since it is now accepted that mRNA levels do not equate with protein concentrations [1], and they reveal nothing at all about post-translational modifications and protein-protein interactions. For information about the expression of the proteome, protein and peptide arrays are becoming major tools and the information that will be obtained from them in the future will complement that from DNA arrays (some recent reviews are listed at the end of this article).

The technology of protein arrays is in many respects an extension of well established methods, such as ELISA and dot blotting, with the new gloss that high throughput detection systems enable the user to investigate thousands of proteins in parallel. One goal, therefore, is to create protein arrays that contain very large sets of proteins and, at the limit, entire proteomes. Protein arrays are also flagged as being the next stage for proteomics. Two-dimensional gel electrophoresis technology, on which most proteome profiling is based [2], is limited in various ways (it is technically demanding, has problems finding proteins present in really low amounts, etc.) and could not be used as a practical diagnostic method. A protein 'chip' could sensitively detect low levels of proteins with minimal technical know-how on the part of the user and be used to measure protein or analyte concentrations in plasma or tissues.

At least three areas for the application of protein arrays can be highlighted, each of which will require different formats and readout methods. They can be summarised as: finding what is present in a protein mixture, such as a tissue extract, and making quantitative comparisons between samples; selecting individual members from libraries of phenotype-genotype linkages for further expression or manipulation; and discovering new functions for proteins, including their interactions, in order to understand the workings of the living cell.

First, there is the detection of proteins in cells and tissue extracts, with application in expression profiling and proteome analysis. A major format will be arrays of immobilised antibodies [3] or ligand-binding scaffolds [4] which will be used to detect quantitative

Copyright © 2001 John Wiley & Sons, Ltd.

differences between samples from healthy and diseased tissues, providing complementary information to mRNA profiling using DNA chips. For differential display, the antibody array could be probed with fluorescently labelled proteins from two different cell states, in which cell lysates are labelled with different fluorophores and mixed such that the colour acts as a readout for the change in abundance [3]. Antigen arrays will find diagnostic applications for detecting antibodies in serum as a screen for infections and autoimmunity.

Secondly, as a two-dimensional display of individual elements, a protein array can be used to screen phage or ribosome display libraries, in order to select specific binding partners, including antibodies [5], synthetic scaffolds, peptides and aptamers. In this way, 'library against library' screening can be carried out, with one library distributed as the immobilised elements of the array and the other being a mixture of protein-gene elements in suspension to which the array is exposed. Drug screening against an array of protein targets identified from genome projects is another possible use.

Thirdly, the biochemical functions of arrayed proteins, such as enzyme activities [6] or interactions with other proteins, DNA, etc. [7], can be tested in parallel. For detecting interactions, protein arrays could be *in vitro* alternatives to the cell-based yeast two-hybrid system and useful where the latter is deficient, such as those interactions involving secreted proteins, proteins with disulphide bridges and membrane-bound proteins.

There are several important technical challenges and bottlenecks in protein array technologies, some of which are unique to proteins while others are common to high throughput methods in general. They include the problems of obtaining global protein expression for array construction, chemical aspects of protein coupling to surfaces, the sensitivity of detection systems, and matters of standardisation and data storage. The issues which need to be addressed include the following:

(i) The limiting step in creating protein arrays, especially those which aim to be global, is the production of the huge diversity of proteins which will form the array elements. Methods available include purification from natural sources [7], cell-based expression systems for recombinant proteins [8], production *in vitro* by cell-free translation systems [9], and synthetic methods (for peptides) [10]. The problem is how to make any of these sufficiently comprehensive such that potentially all proteins become available; expression systems will have to be improved and different systems may have to be used for different proteins.

(ii) Proteins are complicated by the existence of frequent and varied post-translational modifications (PTMs), which increase the number of possible proteins at least 10-fold over the number of genes. The problem will be how to incorporate PTMs into protein arrays.

(iii) Ideally, arrayed proteins should be correctly folded and functional. How can this be ensured and validated?

(iv) How will membrane proteins, which are often functional only in a lipid environment, be arrayed and interrogated on a chip surface?

(v) What are the best coupling chemistries and supports? There are several options available in both categories. For example, support surfaces may be chemically derivatised glass slides [11], agarose films on glass [12], gel pads [13], microwells [14], nitrocellulose [7] or PVDF membranes [8]. What are their relative merits and what is the stability and lifetime of protein arrays in different formats?

(vi) Detection methods are another important consideration, with requirements of sensitivity, accuracy and quantitation over a wide range. Current options include fluorescence [3], mass spectrometry, and DNA amplification (PCR, rolling circle) [15]. The design of the array will be influenced by the readout system.

(vii) Standardisation is an issue which is common to high throughput technologies: the existence and development of many alternative formats and conditions inevitably leads to problems in comparison of results. Standards for protein arrays and a framework for their implementation will need to be established at an international level.

(viii) Data management and databases: how will protein array data be stored, annotated and accessed?

(ix) How will protein interactions detected on chips be interpreted and validated in terms of functional intracellular networks?

(x) Last, but not least: what will be the unit costs of any of the methodologies on offer? Will it be economically feasible to produce proteome chips on the scale of DNA chips?

Copyright © 2001 John Wiley & Sons, Ltd.

### References

1. Gygi SP, Rochon Y, Franza BR, Aebersold R. 1999. Correlation between protein and mRNA abundance in yeast. *Mol Cell Biol* **19**: 1720–1730.
2. Wilkins MR, Williams KL, Appel RD, Hochstrasser DF (eds). 1997. *Proteome Research: New Frontiers in Functional Genomics.* Springer Verlag, Berlin.
3. Haab BB, Dunham MJ, Brown PO. 2001. Protein microarrays for highly parallel detection and quantitation of specific proteins and antibodies in complex solutions. *Genome Biol* **2**: 0004.1–0004.13.
4. Nygren PA, Uhlen M. 1997. Scaffolds for engineering novel binding sites in proteins. *Curr Opin Struct Biol* **7**: 463–9.
5. de Wildt RM, Mundy CR, Gorick BD, Tomlinson IM. 2000. Antibody arrays for high-throughput screening of antibody–antigen interactions. *Nature Biotechnol* **18**: 989–994.
6. Martzen MR, McCraith SM, Spinelli SL, *et al.* 1999. A biochemical genomics approach for identifying genes by the activity of their products. *Science* **286**: 1153–1155.
7. Ge H. 2000. UPA, a universal protein array system for quantitative detection of protein–protein, protein–DNA, protein–RNA and protein–ligand interactions. *Nucleic Acids Res* **28**: e3.
8. Bussow K, Cahill D, Nietfield W, *et al.* 1998. A method for global protein expression and antibody screening on high-density filters of an arrayed cDNA library. *Nucleic Acids Res* **26**: 5007–5008.
9. He M, Taussig MJ. 2001. Single-step generation of protein arrays from DNA by cell free expression and *in situ* immobilisation (PISA method). *Nucleic Acids Res* **29**: e73.
10. Reinecke U, Volkmer-Engert R, Schneider-Mergener J. 2001. Applications of peptide arrays prepared by the SPOT-technology. *Curr Opin Biotechnol* **12**: 59–64.
11. MacBeath G, Schreiber SL. 2000. Printing proteins as microarrays for high-throughput function determination. *Science* **289**: 1760–1763.
12. Afanassiev V, Hanemann V, Wölfl S. 2000. Preparation of DNA and protein microarrays on glass slides coated with an agarose film. *Nucleic Acids Res* **28**: e66.
13. Arenkov P, Kukhtin A, Gemmell A, Voloshchuk S, Chupeeva V, Mirzabekov A. 2000. Protein microchips: use for immunoassay and enzymatic reactions. *Anal Biochem* **278**: 123–131.
14. Zhu H, Klemic JF, Chang S, *et al.* 2000. Analysis of yeast protein kinases using protein chips. *Nature Genet* **26**: 283–289.
15. Schweitzer B, Wiltshire S, Lambert J, *et al.* 2000. Immunoassays with rolling circle DNA amplification: a versatile platform for ultrasensitive antigen detection. *Proc Natl Acad Sci U S A* **97**: 10113–10119.

### Recent reviews on protein arrays

Cahill DJ. 2001. Protein and antibody arrays and their medical applications. *J Immunol Methods* **250**: 81–91.

Grayhack EJ, Phizicky EM. 2001. Genomic analysis of biochemical function. *Curr Opin Chem Biol* **5**: 34–39.

Holt LJ, Enever C, de Wildt RM, Tomlinson IM. 2000. The use of recombinant antibodies in proteomics. *Curr Opin Biotechnol* **11**: 445–449.

Kodadek T. 2001. Protein microarrays: prospects and problems. *Chemistry and Biology* **8**: 105–115.

Tomlinson IM, Holt LJ. 2001. Protein profiling comes of age. *Genome Biol* **2**: 1004.1–1004.3.

Walter G, Bussow K, Cahill D, Lueking A, Lehrach H. 2000. Protein arrays for gene expression and molecular interaction screening. *Curr Opin Microbiol* **3**: 298–302.

Zhu H, Snyder M. 2001. Protein arrays and microarrays. *Curr Opin Chem Biol* **5**: 40–45.

Copyright © 2001 John Wiley & Sons, Ltd. 
